# Optimisation of Over-Expression in *E. coli* and Biophysical Characterisation of Human Membrane Protein Synaptogyrin 1

**DOI:** 10.1371/journal.pone.0038244

**Published:** 2012-06-01

**Authors:** Christian Löw, Caroline Jegerschöld, Michael Kovermann, Per Moberg, Pär Nordlund

**Affiliations:** 1 Department of Medical Biochemistry and Biophysics, Karolinska Institutet, Stockholm, Sweden; 2 Department of Biosciences and Nutrition, Karolinska Institutet, Huddinge, Sweden; 3 Institut für Physik, Biophysik, Martin-Luther-Universität Halle-Wittenberg, Saale, Germany; 4 School of Biological Sciences, Nanyang Technological University, Singapore, Singapore; Aligarh Muslim University, India

## Abstract

Progress in functional and structural studies of integral membrane proteins (IMPs) is lacking behind their soluble counterparts due to the great challenge in producing stable and homogeneous IMPs. Low natural abundance, toxicity when over-expressed and potential lipid requirements of IMPs are only a few reasons for the limited progress. Here, we describe an optimised workflow for the recombinant over-expression of the human tetraspan vesicle protein (TVP) synaptogyrin in *Escherichia coli* and its biophysical characterisation. TVPs are ubiquitous and abundant components of vesicles. They are believed to be involved in various aspects of the synaptic vesicle cycle, including vesicle biogenesis, exocytosis and endocytotic recycling. Even though TVPs are found in most cell types, high-resolution structural information for this class of membrane proteins is still missing. The optimisation of the N-terminal sequence of the gene together with the usage of the recently developed Lemo21(DE3) strain which allows the balancing of the translation with the membrane insertion rate led to a 50-fold increased expression rate compared to the classical BL21(DE3) strain. The protein was soluble and stable in a variety of mild detergents and multiple biophysical methods confirmed the folded state of the protein. Crosslinking experiments suggest an oligomeric architecture of at least four subunits. The protein stability is significantly improved in the presence of cholesteryl hemisuccinate as judged by differential light scattering. The approach described here can easily be adapted to other eukaryotic IMPs.

## Introduction

Integral membrane proteins (IMPs) are notoriously difficult to study. The lack of structural data is just one consequence of the challenges they confront us with: toxicity to their over-production host, lipid requirements for correct folding and function, detergents destabilising the IMPs and hampering the formation of well-ordered crystals [1].

Since the natural abundance of IMPs is usually low, they need to be over-expressed in a recombinant system. To be able to isolate sufficient material for structural and functional studies the choice of the over-expression host is critical [2]. Bacteria such as *Escherichia coli* are preferred since they are well studied, genetically accessible and can produce a large biomass in time and cost effective manner [3], [4]. As a consequence, the *E. coli* BL21(DE3) strain is the most widely used protein production system. In BL21(DE3) the expression of the target gene is directed by the T7 RNA polymerase [5], a polymerase 8 times faster than the *E. coli* RNA polymerase [6]. The strain was designed for rapid and efficient protein production [5]. However, over-expression of IMPs is often toxic to cells. A too fast transcription/translation rate seems to lead to saturation of the bacterial membrane protein insertion machinery, the Sec translocon [7], [8]. Therefore an *E. coli* strain, Lemo21(DE3), was recently engineered to balance expression speed with the cell's capacities by titrating the T7 RNA polymerase activity [9]. In practice, the production of IMPs in a homogeneous and functional form still requires time consuming screening of many different conditions.

Here we present a comprehensive over-expression optimisation screening strategy and the biophysical characterisation of a human IMP over-expressed in *E. coli*. The strategy has been applied for synaptogyrin, an IMP which belongs to the tetraspan vesicle membrane proteins (TVPs). TVPs have four transmembrane segments and cytoplasmically located termini, and are highly abundant components of different vesicle types [10]–[13]. They can be grouped into three distinct families that are referred to as physins, gyrins and secretory carrier associated membrane proteins (SCAMPs). They are encoded by multigene classes in mammals and are evolutionary conserved throughout the animal kingdom [10], [14]. Numerous roles of TVPs in various aspects of the (synaptic) vesicle cycle, including vesicle biogenesis, exocytosis and endocytotic recycling [10], [14] are suggested. Furthermore, multiple interactions of TVPs with lipids (mainly cholesterol) [15], [16], the dopamine transporter [17] and various components of the recycling machinery like the soluble N-ethylmaleimide sensitive fusion attachment protein receptors and syntaxin (SNAREs) [18]–[21], dynamin [22]–[24], eps15 homology (EH)-domain proteins [25] have been described. The observation of mild or even absent phenotypic defects in neurons of mice lacking synaptogyrin 1, synaptophysin or SCAMP 1 is intriguing [14], [26]–[30]. Combined knockouts of the genes encoding synaptophysin and synaptogyrin lead to changes in synaptic plasticity in mice, whereas a triple mutant (synaptogyrin 1, synaptophysin 1, SCAMP 1) in *Caenorhabditis elegans* is lacking profound nervous system defects [14].

A number of mutations, insertions and deletions of the gene encoding synaptogyrin 1 were identified in schizophrenia patients suggesting that aberrant synaptogyrin 1 function may be involved in the pathogenesis of schizophrenia [31]–[34]. Despite these findings the understanding of synaptogyrin in the synaptic vesicle cycle is far from complete. To study the structural and functional role of synaptogyrin further we have established an over-expression platform for different synaptogyrin isoforms. The combination of synaptogyrin construct optimisation and the usage of the recently described *E. coli* strain Lemo21(DE3) improved expression levels from micrograms to several milligrams per liter of *E. coli* culture, sufficient for structural studies. A number of biophysical techniques validated that the recombinant expressed synaptogyrin 1 was homogeneous and properly folded. Crosslinking experiments support an oligomeric assembly in the membrane and in detergent solubilised form with at least four subunits. The purified protein is significantly stabilised against heat denaturation in the presence of cholesteryl hemisuccinate. The strategy for improving eukaryotic IMP over-expression in *E. coli* provided here can easily be adapted to other IMPs.

## Results

### Cloning and construct design

**Figure 1:**
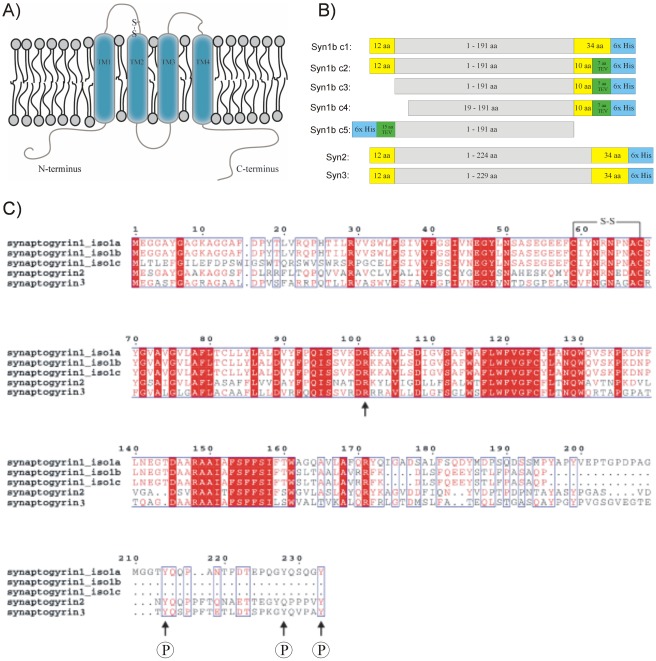
Topology model, construct design and sequence comparison of synaptogyrin. (A) Topology model and schematic representation of human TVP synaptogyrin in the vesicle membrane. Both, N- and C-termini are predicted to face the cytoplasm. Transmembrane domains are indicated. (B) Synaptogyrin constructs designed for this over-expression study. Additional amino acid residues and affinity tags provided by the vector backbone are color coded. (C) Sequence comparison of the synaptogyrin members 1–3 including isoforms a-c from synaptogyrin 1. Identical and similar residues are color coded in red. The disulfide bridge in vesicular loop 1, a highly conserved arginine in loop 2 and possible phosphorylation sites in the C-terminal part are indicated. Synaptogyrin members show greatest variability in the cytoplasmic C-terminal tail. Synaptogyrin 1 isoforms b and c lack possible phosphorylation sites.

The schematic representation of the synaptogyrin family is shown in figure 1A [10], [35]–[39]. All synaptogyrin members are composed of a short N-terminal cytoplasmic sequence, four homologous transmembrane segments and a variable cytoplasmic C-terminal tail that is tyrosine phosphorylated (number and position arbitrary). A 38 amino acid stretch within the C-terminal region of synaptogyrin 1 and a single arginine residue in the cytoplasmic loop 2 are required for its correct targeting [37]. Paired cysteines are present in the intravesicular loop 1 of all family members. The high sequence similarity between synaptogyrin isoforms is mainly due to the highly conserved transmembrane domains, whereas major differences appear in the N- and C-terminal residues (Figure 1C). Furthermore, the splice forms synaptogyrin 1b and 1c have shortened C-termini compared to synaptogyrin 1a, synaptogyrin 2 and synaptogyrin 3, which are predicted to be in random coil conformations [40]. Synpatogyrin 1b, 2 and 3 were chosen for a detailed over-expression study in *E. coli*. The genes encoding the different synaptogyrin variants were initially cloned into the pTH24 vector using the Gateway cloning system from the human Orfeome collection (see “Materials and Methods” section for details). This robust and high throughput compatible cloning system however adds additional 12 residues N-terminal and 40 residues C-terminal of the target protein which is not desired for structural studies. Therefore additional N-terminal deletion constructs of synaptogyrin 1b were designed as shown in Figure 1B. The synaptogyrin 1b gene was shortened from the N-terminus and a TEV cleavage site was introduced in front of the C-terminal His tag. Furthermore construct 5 of synaptogyrin 1 contained a cleavable N-terminal His tag and no additional residues at the C-terminus.

### Expression screening

**Figure 2:**
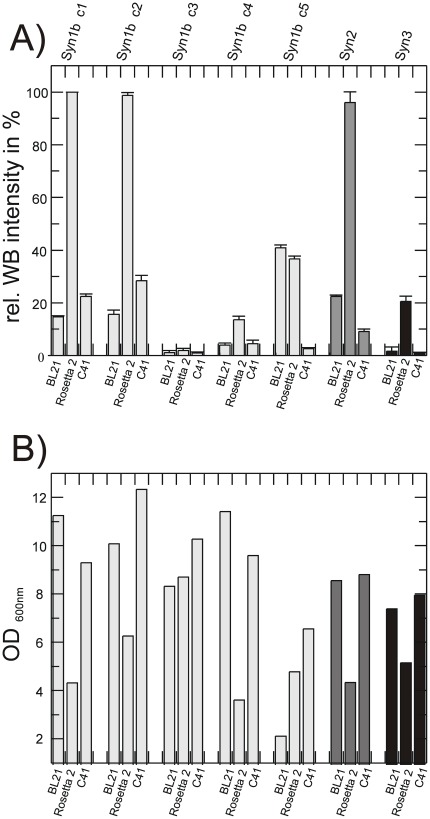
Over-expression of synaptogyrin constructs. (A) Relative over-expression yields of the synaptogyrin constructs in different expression strains determined *via* Western blot after crude membrane preparation using a HRP-conjugated His probe. Construct with the highest Western blot intensity was set to 100 percent. Error bars are derived from three independent Western blots to minimise errors caused by inefficient transfer of IMPs from the SDS gels onto the membrane. (B) Final optical density of the synaptogyrin constructs in the corresponding expression strain prior to harvest.

**Figure 3:**
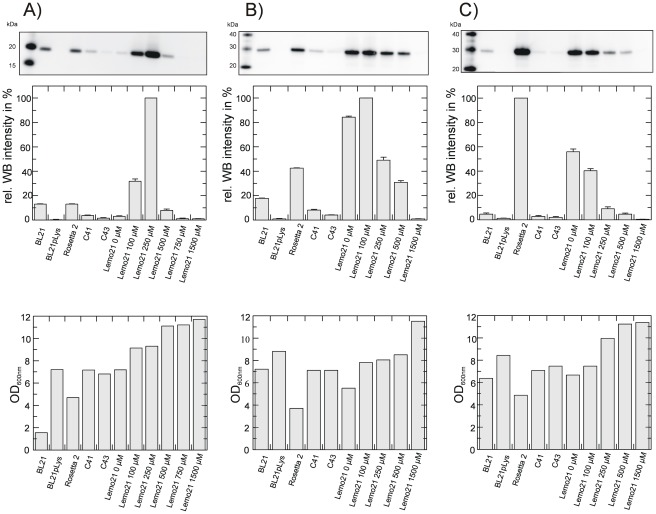
Lemo21(DE3) as over-expression host for synaptogyrin. (A) Over-expression yields of synaptogyrin 1b construct 5 as quantified by Western blot of crude membranes in different over-expression strains. The different rhamnose concentrations used with the Lemo21(DE3) strain are indicated. Expression strain with the highest expression level was set to 100 percent Western blot intensity. Lower panel shows the final OD_600 nm_ values reached before harvest. (B) Same as in (A) but data are shown for the synaptogyrin 2 construct. (C) Over-expression results for synaptogyrin 3 as described for (A). Error bars are derived from three independent Western blot analyses.

Three different *E. coli* strains (BL21(DE3), C41(DE3) and Rosetta2(DE3)) were chosen for initial expression screening. Expression trials revealed very low expression levels of the IMPs at induction temperatures above 25°C (data not shown). Highest expression yields were obtained at 20°C and cells were harvested 16 hours after induction. Prepared membranes were normalised according to the final OD_600 nm_ value and expression levels were quantified *via* Western blot analysis (Figure 2A). Expression levels of the synaptogyrin 1 constructs varied significantly. The Rosetta2(DE3) strain performed best although the obtained biomasses of the different over-expression cultures, reflected in the lowest OD_600 nm_ values, were the lowest (Figure 2B). Most dramatic reduction of the expression levels for synaptogyrin 1 was caused by removing the 12 additional N-terminal residues provided by the plasmid backbone (compare construct 1 and construct 3), an observation we have made for a number of IMPs (unpublished results). However, placing a codon optimised His tag at the N-terminus including a TEV cleavage site (construct 5) yielded 40% of expression level compared to construct 1 and 2. This construct is preferred for structural studies since only one additional residue remains on the protein after removal of the His tag. Based on the large differences of over-expression performances in different *E. coli* strains we extended the expression screening repertoire by the second “Walker strain” C43(DE3) [41] and the newly developed Lemo21(DE3) expression system [9]. The latter controls the activity of T7 RNA polymerase by its natural inhibitor, the T7 lysozyme, which is encoded on an additional plasmid under the control of a titratable rhamnose promoter. With this system it is possible to balance expression rate with membrane insertion capacity to minimise potential toxic effects of membrane protein over-expression and thus to increase the overall expression yield. Indeed, dependent on the target proteins, different rhamnose concentrations were identified for optimal expression levels [9] (Figure 3). For the N-terminal His tagged construct of synaptogyrin 1b (construct 5) an 8-fold increase in expression level per cell compared to the best expressing *E. coli* strain Rosetta2(DE3) was found for Lemo21(DE3) in the presence of 250 µM rhamnose (Figure 3A). Expression levels in the presence of lower rhamnose concentrations are reduced probably due to the jamming of the translocon. Higher rhamnose concentrations presumably lead to too low activity of the T7 RNA polymerase resulting in reduced yields. Surprisingly, none of the “Walker strains”, C41(DE3) and C43(DE3), originally isolated for their improved membrane protein over-expression characteristics showed increased expression levels for any of the synaptogyrin constructs. The effects for synaptogyrin 2 and 3 were less dramatic (Figure 3B,C). While synaptogyrin 2 showed an 2-fold increase in expression in Lemo21(DE3), the Rosetta2 strain remained the best over expression host for synaptogyrin 3. Besides the increased expression levels per cell for synaptogyrin 1 and 2, the resulting biomass reflected in the higher OD_600 nm_ values was also significantly increased for the Lemo21(DE3) system compared the Rosetta2(DE3) strain. Overall, an almost 20-fold increase in total synaptogyrin 1 (construct 5) over-expression level was obtained.

### Detergent solubilisation screening and quality control

**Figure 4:**
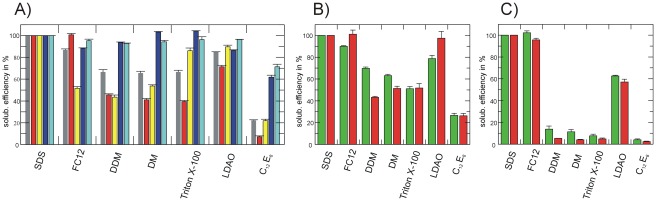
Detergent screening of synaptogyrin constructs. (A) Solubilisation efficiencies of synaptogyrin 1 constructs in different detergents (SDS, FC12, DDM, DM, Triton X-100, LDAO and C_12_E_8_). Crude membranes solubilised in SDS were used as a reference and obtained Western blot signal corresponds to 100 percent solubilisation efficiency. Only membranes with the highest expression levels for a particular constructs were used for this solubilisation screen: (**−**) syn1b c1 (Rosetta2(DE3)); (**−**) syn1b c2 (Rosetta2(DE3)); (**−**) syn1b c4 (Rosetta2(DE3)); (**−**) syn1b c5 (Rosetta2(DE3)); (**−**) syn1b c5 (Lemo21(DE3), 250 µM rhamnose). (B) Comparison of solubilisation efficiencies for the synaptogyrin 2 protein expressed in the (**−**) Rosetta2(DE3) strain or (**−**) Lemo21(DE3) (100 µM rhamnose). (C) Relative solubilisation efficiencies of the synaptogyrin 3 protein expressed in the (**−**) Rosetta2(DE3) strain or (**−**) Lemo21(DE3) (0 µM rhamnose). Error bars are derived from three independent Western blot analyses.

The expression levels for the synaptogyrin variants in different *E. coli* strains were determined after membrane preparations indicating that the recombinantly expressed IMP is either inserted or associated with the membrane fraction. This however does not give any information on the quality of the over-expressed material. In contrast to soluble proteins, where incorrectly folded proteins usually precipitate and end up as inclusion bodies, the situation for IMPs is different: Over-expressed IMPs can be targeted to and inserted into or associated with the membrane, which does not automatically ensure that the protein has reached the native folded state. Initial quality information can be obtained by solubilisation screening. Prepared membranes from *E. coli* strains showing the highest expression level for synaptogyrin 1, 2 and 3 according to Western blot analysis, were solubilised in eight different detergents generally used in IMP biochemistry and the solubilisation efficiency was subsequently quantified (for details see “Materials and Methods” section). High solubilisation efficiencies in mild detergents such as DDM, DM, C_12_E_8_ or Triton X-100 are already a good indication for a properly folded IMP. On the other hand, if solubilisation is only successful in harsh detergents such as FC12 or LDAO, which are able to solubilise precipitated material [42], there is a high risk that the IMP is not functionally folded in the membrane. Interestingly, the solubilisation efficiencies vary for different synaptogyrin isoforms and constructs (Figure 4). Just about 50% of the over-expressed material of synaptogyrin 1 constructs with a C-terminal His tag could be solubilised in mild detergents, while the N-terminally tagged constructs expressed either in the Rosetta2(DE3) or Lemo21(DE3) strain was almost 100% soluble in all detergents tested (Figure 4A). Observations for the synaptogyrin 2 construct are very similar compared to synaptogyrin 1 (Figure 4B). For synaptogyrin 3 the situation is different: Less than 15% of the over-expressed material was soluble in detergents other than FC12 or LDAO indicative of poorly folded and instable material (Figure 4C).

**Figure 5:**
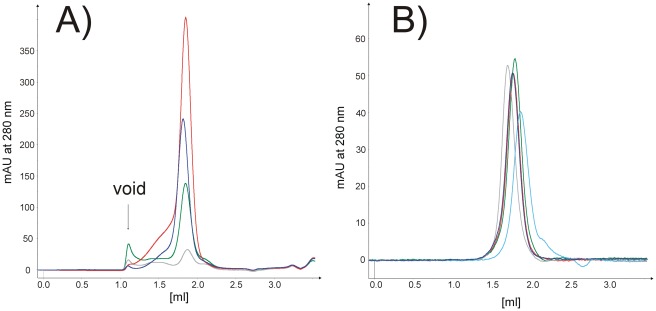
Analytical gel filtration (AGF) screening of synaptogyrin. (A) Analytical gel filtration of different synaptogyrin 1 constructs after solubilisation in DDM and subsequent IMAC purification. The void volume is indicated. Synaptogyrin 1 constructs elute as monodisperse peaks from the gel filtration column indicative for high homogeneity: (**−**) syn1b c1; (**−**) syn1b c2; (**−**) syn1b c4; (**−**) syn1b c5. (B) Synaptogyrin 1b construct 5 remains stable and elutes as symmetrical peak from the analytical gel filtration column in different detergents ((**−**) DDM; (**−**) UDM; (**−**) DM; (**−**) LDAO; (**−**) OG).

To characterise the solubilised material further, the different DDM solubilised synaptogyrin 1 constructs were purified *via* affinity chromatography and analysed by analytical gel filtration (Figure 5). Synaptogyrin 1 constructs elute as a monodisperse symmetrical peak typical for a homogeneous protein preparation (Figure 5A). Based on the high quality and expression level in Lemo21(DE3) the N-terminally His tagged synaptogyrin 1 construct was used for further evaluation (construct 5). As already indicated in the solubilisation screen, synaptogyrin 1 could be solubilised in various detergents and remained monodisperse during the gel filtration run (Figure 5B).

**Figure 6:**
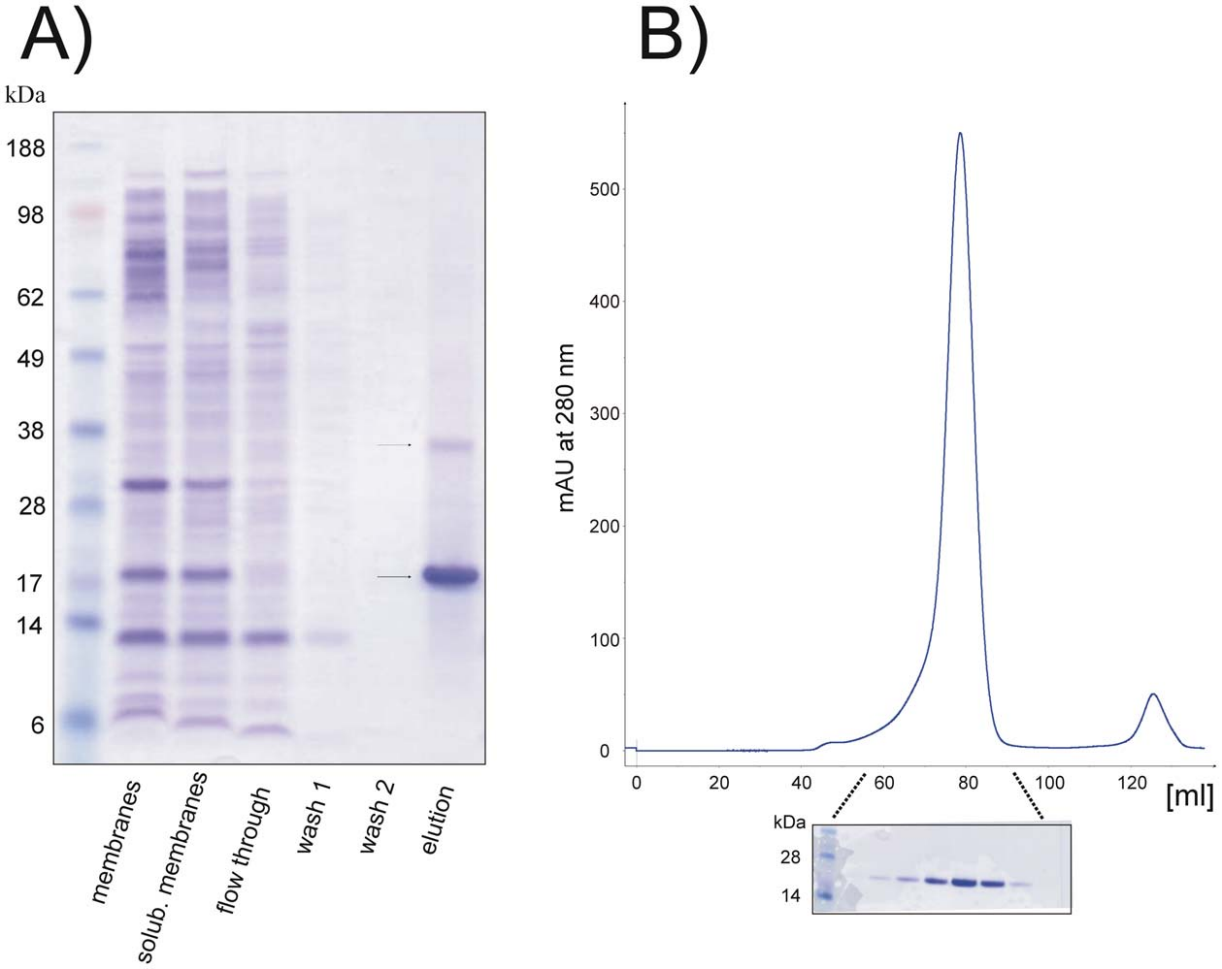
Large scale expression of synaptogyrin 1. (A) SDS-PAGE from large scale purification of synaptogyrin 1b construct 5 over-expressed in the Lemo21(DE3) strain in the presence of 250 µM rhamnose. High level expression and efficient IMAC beads binding results in a high yield of recombinantly produced synaptogyrin 1. A potential dimeric form is indicated. (B) The IMAC purified protein elutes as a single monodisperse peak from a preparative gel filtration column.

Results obtained from the small-scale expression screening translated very well in large scale expression and purification as shown in Figure 6. Five milligrams of purified synaptogyrin 1 were obtained per liter of culture after IMAC chromatography and preparative gel filtration. The protein remained stable without any obvious precipitation at room temperature for one week and could be concentrated above 20 mg/ml.

### Oligomeric state of synaptogyrin 1

**Figure 7:**
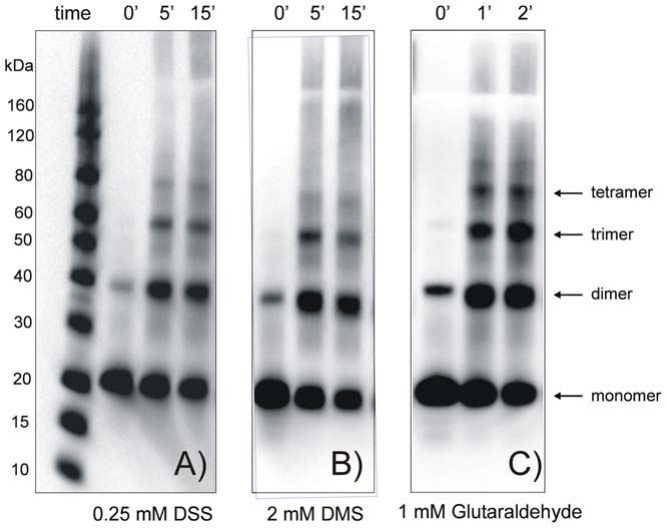
Crosslinking experiments of synaptogyrin 1 in the presence of different bifunctional crosslinkers support an oligomeric architecture. (A) Crosslinking of DDM solubilised and purified synaptogyrin 1 in the presence of 0.25 mM DSS. (B) Higher oligomeric species of synaptogyrin 1 are also obtained when the crosslinking reaction (2 mM DMS) is performed on crude membranes. (C) Similar conclusions result from crosslinking experiments of Triton X-100 solubilised and purified material in the presence of 1 mM glutaraldehyde. Crosslinking reactions were stopped after the given time points by adding 200 mM of Tris solution and subsequent boiling of the sample.

**Figure 8:**
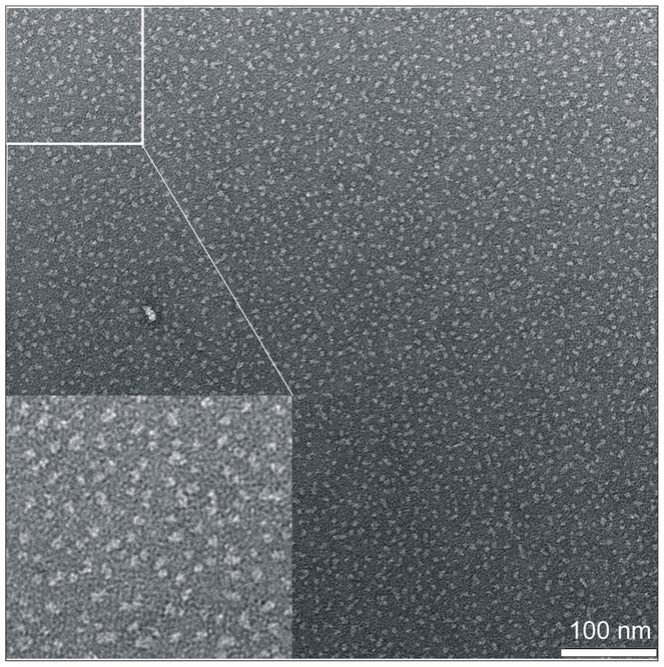
Negative-stain TEM of single purified synaptogyrin 1 protein in the presence of the detergent DDM. The homogeneity of the purified protein is reflected in the electron micrograph. Synaptogyrin particles have a diameter of around 5–6 nm. The scale bar represents 100 nm. Magnifications are shown.

TVPs are predicted to form oligomers of four to six monomers. An SDS resistant dimeric form of synaptogyrin 1 already indicated that synaptogyrin 1 exists as a higher oligomer (Figure 6A). Crosslinking experiments of synaptogyrin 1 in native membranes and in its solubilised form (DDM and Triton were used as detergents) with various crosslinkers suggest at least a tetrameric assembly (Figure 7A, B, C). This is further supported by electron microscopy. Synaptogyrin 1 preparations in DDM (Figure 8) are highly homogenous without any obvious aggregation/precipitation. Considering the membrane thickness of about 4–5 nm even the smallest transmembrane protein need to show such a dimension which is also the case here. The particle size of 5–6 nm argues against oligomers higher than tetramers. Furthermore, the small, featureless shape indicates tight bundles of α-helices as opposed to the 12–14 helices in the transporter DtpD that displays an open crown like shape [43].

### NMR and CD spectroscopy of synaptogyrin 1

**Figure 9:**
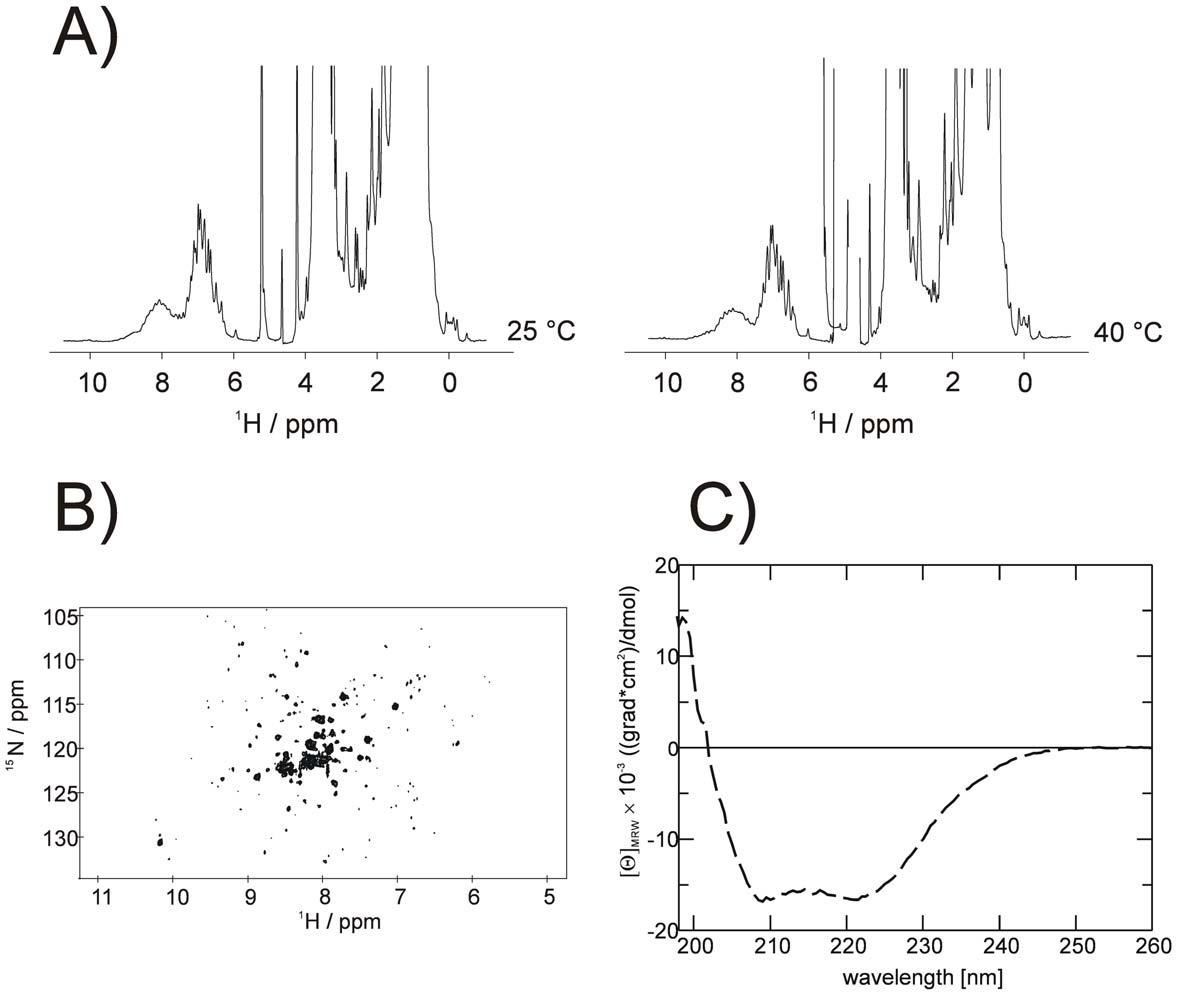
NMR- and CD-spectroscopy of synaptogyrin 1. (A) One-dimensional proton NMR spectra at two different temperatures as well as the (B) heteronuclear ^1^H^15^N TROSY-HSQC spectrum show a well dispersed amid proton signal region (^1^H = 6 to 9 ppm). The high field shifted aliphatic resonance signals (^1^H<0 ppm) strongly support a well folded protein state for synaptogyrin 1. (C) Far UV-CD spectrum of synaptogyrin 1 indicates a high degree of alpha helical secondary structure with more than 50 percent of the residues being in an alpha helical conformation.

To further explore the folding state of the purified detergent solubilised protein, we used NMR and CD spectroscopy (Figure 9). The far UV-CD spectrum of synaptogyrin 1 (construct 5) reveals the expected helical fold with more than 50% of the residues being in an alpha helical conformation (Figure 9C). The one-dimensional proton spectra as well as the heteronuclear ^1^H-^15^N TROSY-HSQC spectra show a reasonable dispersion within the amid proton signal region (^1^H = 6 to 9 ppm, for the whole temperature range used; Figure 9A,B). The very high field shifted aliphatic resonance signal (^1^H = −0.4 ppm at 25 and 40°C, Figure 9A) strongly indicates that synaptogyrin 1 is well folded. Stability of synaptogyrin 1 was tested by heating the protein sample up to 45°C and subsequent cooling to 25°C. Spectra before and after heat treatment were virtually identical (data not shown), indicating sufficient stability for structural studies.

### Stability of synaptogyrin 1

**Figure 10:**
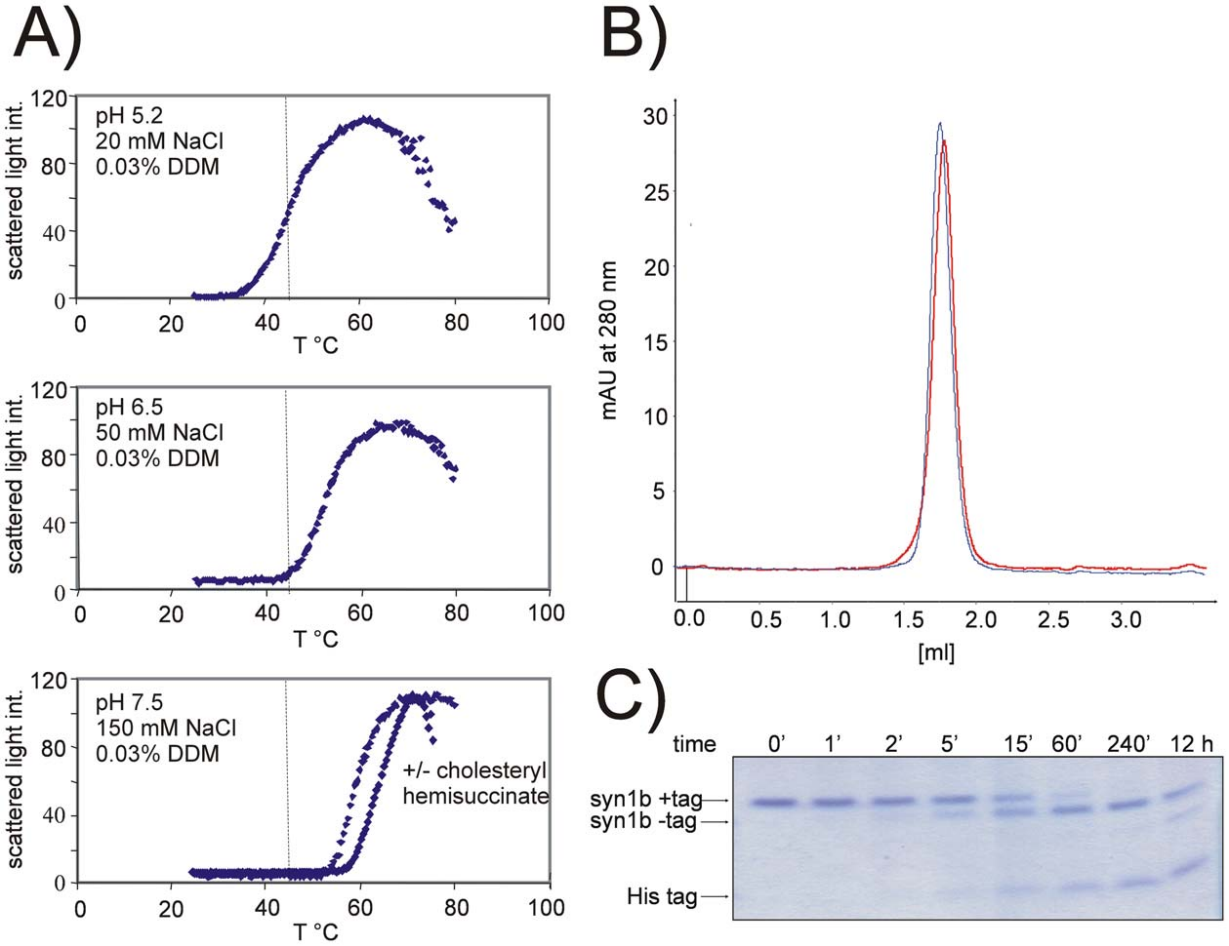
Stability dependence of synaptogyrin 1. (A) Thermal stability of synaptogyrin 1 was followed *via* differential static light scattering (DSLS) in a 384 well plate format. The protein is most stable against heat denaturation at neutral pH and physiological concentrations of NaCl. Examples of unfolding curves in three different conditions are shown. The stability is further enhanced in the presence of cholesteryl hemisuccinate. (B) The analytical gel filtration profile is not significantly altered in the presence of 0.03 mg/ml brain lipids and 0.005% cholesteryl hemisuccinate. (**−**) syn1b in 20 mM sodium phosphate, 150 mM NaCl, 5% glycerol, 0.03% DDM (**−**) syn1b in 20 mM sodium phosphate, 150 mM NaCl, 5% glycerol, 0.03 mg/ml brain lipids, 0.03% DDM, 0.005% cholesteryl hemisuccinate. (C) Limited proteolysis in the presence of chymotrypsin: Synaptogyrin 1 (construct 5) is stable against proteolytic degradation at a protein to protease ratio of 100∶1. Only the N-terminal His tag is cleaved as verified by mass spectrometry.

To assess the thermal stability of synaptogyrin 1 we used differential static light scattering (DSLS) in a 384 well format [44]. Upon unfolding, proteins, including IMPs, have a high tendency for precipitation resulting in an increased light scattering signal. Light scattering of synaptogyrin 1 in a variety of conditions (pH, salt, detergents, lipids, and cholesteryl hemisuccinate) was followed at increasing temperatures between 25 and 80°C. Figure 10A shows that synaptogyrin 1 is significantly stabilised at neutral pH and physiological concentrations of salt. The protein is further stabilised upon addition of cholesteryl hemisuccinate. Whether there is a direct interaction of cholesterol with synaptogyrin needs to be shown, but synaptic vesicles are rich in cholesterol [13] and cholesterol interaction has been proposed for the TVP synaptophysin [15], [16]. The presence of cholesteryl hemisuccinate does neither significantly alter quality nor oligomeric state of the purified protein as shown by analytical gel filtration experiments (Figure 10B). Characteristic for eukaryotic proteins are often disordered N- and C-terminal regions, which can hamper crystallisation significantly. To address this issue, we used limited proteolysis to study whether synaptogyrin 1 is susceptible for proteolytic degradation. The entire protein remains stable and folded in the presence of high concentrations of chymotrypsin. Only the N-terminal His tag is cleaved within a short time period as verified by mass spectrometry (Figure 10C).

## Discussion

Despite their likely high importance in synaptic vesicles, the elucidation of the functional role of TVPs remains challenging. The problems with elucidating the exact cellular function is partly due to the lack of phenotypes in knockout mice and *C. elegans* [14], [26]–[30]. To gain deeper insights into structure and functional properties of human synaptogyrin we established the recombinant production of homogeneous material in *E. coli*. In spite of that relatively few human IMPs have previously been over-expressed in the bacterial host *E. coli*, this study shows that screening parameters such as construct design and bacterial expression strain can be the key for successful production of high quality material of human IMPs. Compared to the classical *E. coli* BL21(DE3) strain an almost 50-fold increase in homogeneous material per liter of culture could be obtained for synaptogyrin 1 in the Lemo21(DE3) strain taking the increased expression level per cell and biomass into account. Results of our initial over-expression experiments pointed towards an important role of the conflicting codon usage between the target gene and the bacterial host. The *E. coli* strain Rosetta2(DE3) provides tRNAs for rare codons in order to enhance the translation efficiency of “foreign” genes. It performed best for all initial constructs of the different synaptogyrin isoforms tested. However, removing the codon optimised N-terminal residues of the construct provided by the vector backbone (compare construct 1–4, Figure 1) almost completely abolished over-expression. Replacing these 12 additional N-terminal residues by a codon optimised His tag including a TEV cleavage site restored almost 40% of the original expression level. Therefore it seems more important to optimise the codon usage for the N-terminal residues than for the whole gene to guarantee an efficient translation start [45]. Based on previous over-expression studies and the work presented here, slower translation rates are preferred in order to balance expression speed with the insertion process of the IMP into the membrane *via* the Sec translocon (see [3]). So far optimisation of functional IMP over-expression using codon optimised genes in *E. coli* has not been systematically applied. Taking recent findings on translational rate and membrane insertion capacity into account it might even be counterproductive [8], [9]. In order to compare over-expression levels of IMPs expressed under different conditions, or as different constructs, it is essential to analyse the quality and homogeneity of the over-expressed material. Unlike for soluble protein, where incorrectly folded polypeptide chains usually end up in inclusion bodies, IMPs can be targeted to the membrane and end up as precipitated material in the membrane fraction. Indication for this is the absolute requirement for harsh detergents like SDS or FC12 to solubilise the target IMP. The solubilisation efficiencies for synaptogyrin 1 are strongly dependent on the construct used. Almost 100% of the N-terminally tagged synaptogyrin 1 construct can be solubilised in a variety of mild detergents while just half of the over-expressed material for the C-terminally His tagged construct can be extracted in the same detergents. We conclude that the destabilising effect of the detergent upon extraction most likely is not the reason for reduced extraction efficiency. Almost 50% of the C-terminally tagged synaptogyrin polypeptide chains were not properly folded after membrane insertion.

A variety of biophysical methods can be used to analyse the folding state of IMPs. While NMR spectroscopy is a powerful tool and gives more detailed information on sample quality, far UV-CD spectroscopy requires much less purified material but provides only information about the secondary structure of the protein. This analysis tool also should be used with caution, since strong detergents can induce helical structures indicating a properly folded protein state. An example is the far UV-CD spectrum of the beta barrel protein VDAC1, expressed as insoluble material in an *in vitro* expression system and solubilised in the detergent FC12, where it shows the misleading signature of an alpha helical protein, distinct from the native state [46]. Correctly refolded VDAC1 results in a CD spectrum expected for a protein rich in beta sheet structures. The combination of both techniques (NMR- and CD spectroscopy) deployed here for synaptogyrin 1 corroborate the folded state of the recombinately over expressed protein. Synaptogyrin 1 shows an expected alpha helical far UV-CD spectrum and the heteronuclear ^1^H-^15^N TROSY-HSQC spectra has a reasonable dispersion within the amid proton signal region. Although only about 80% of the expected signals are visible, the spectrum has a fingerprint typical for folded proteins. The usage of short chain detergents and the optimization of detergent to protein ratio could help to improve the quality of the spectrum in the future.

To determine the thermodynamic stability of IMPs against chemical or temperature induced denaturation is problematic since almost all IMPs denature in an irreversible manner. However, apparent melting temperatures T_m_s can be determined and used as measure of stability. Comparisons of T_m_s obtained from different experiments have to be done carefully since they depend on factors such as heating rate or protein concentration. Batch to batch variations from IMP preparations resulting in different lipid content and detergent concentration complicate the situation further. Nevertheless, careful measurements of T_m_s of an IMP in a multi-well plate format, as described here, allows for the simultaneously detection of up to 384 conditions in a single experiment, and can provide important information on stabilizing parameters. Synaptogyrin 1 was most stable at neutral pH and physiological concentrations of salt. Shorter chain detergents and higher detergent concentrations reduced the stability, while the addition of a mixture of brain lipids and cholesteryl hemisuccinate stabilised the IMP further. Whether this is due to direct binding of the lipids and cholesteryl hemisuccinate to synaptogyrin 1, needs to be further established, but cholesterol binding to TVPs has been reported previously [15], [16].

Much remains to be learned about the biochemical and physiological roles of synaptogyrin 1. The presented recombinant over-expression, purification and biophysical characterisation of synaptogyrin 1 and 2 provides a solid foundation for further studies on this protein.

## Materials and Methods

### Materials and reagents

IPTG and all detergents were purchased from Affymetrix. Luria-Bertani, Miller (LB) was from Becton Dickinson and Terrific broth (TB) was from Formedium. Carbenicillin and chloramphenicol were from Duchefa. Tween® 20 was from Merck. The *E. coli* strain Lemo21(DE3) was from New England Biolabs. All other chemicals were from Sigma, unless otherwise stated.

### Gene construction

The genes coding for the three different synaptogyrin homologues (Synaptogyrin 1, transcript variant 1b (BC000731), synaptogyrin 2 (BC000407), synaptogyrin 3 (BC009568)) were cloned into a pTH24 vector [47] *via* homologous recombination (Gateway® cloning, Invitrogen) from the hORFeome collection (http://hordb.dfci.hardvard.edu). For cleavage of the C-terminal His tag a Tobacco Etch Virus (TEV) cleavage site was introduced *via* blunt end PCR. For constructs three and four of synaptogyrin 1 nucleotides coding for the N-terminal residues of synaptogyrin were removed by blunt end PCR. For the N-terminal His tag construct (construct 5), the synaptogyrin 1 gene was cloned into the pNIC28-Bsa4 vector using ligation independent cloning [48]. All vectors posses a T7 promoter and terminator sequence. Expression was performed in five different *E. coli* strains: BL21(DE3), BL21(DE3) pLysS, Rosetta2(DE3), C41(DE3), C43(DE3) and Lemo21(DE3). BL21(DE3) is deficient of the Lon protease and is lacking the outer membrane protease OmpT but carries the lambda DE3 lysogen which expresses T7 RNA polymerase from the *lac*UV5 promoter by IPTG induction. The mutant strains C41(DE3) and C43(DE3) were derived from BL21(DE3), selected for their improve over-expression performances of toxic membrane proteins [41]. Rosetta2(DE3) is a host strain that had been transformed with the plasmid pRARE2, which provides tRNAs for seven codons (AGG, AGA, AUA, CUA, CCC, GGA and CGG) rarely used in *E. coli* in order to enhance the translation efficiency of genes with codons different from endogenous *E. coli* ones [49]. Lemo21(DE3) is a BL21(DE3) derivative, in which the activity of the T7 RNA polymerase can be precisely controlled by its natural inhibitor T7 lysozyme [9].

### Small scale protein over-expression

Cultures of 50 ml Terrific Broth (TB) medium in 300 ml baffled conical flasks were inoculated from a Luria Bertani medium (LB) over night culture to a start OD_600 nm_ of 0.05 per ml and grown at 37°C at 200 rpm. At an OD_600 nm_ 0.8–1.0 the temperature was reduced to 20°C over 60 min followed by isopropyl β-D-1-thiogalactopyranoside (IPTG) induction (200 µM). Cultures continued to grow for further 16 hours prior harvest. Cell density was monitored by measuring the OD_600 nm_ value. 40 ml of the cultures were harvested at 5,000 g for six minutes and the pellets were stored frozen at −80°C.

### Membrane preparation

The frozen cell pellet was thawed on ice and resuspended in 15 ml lysis buffer (20 mM sodium phosphate, 300 mM NaCl, pH 7.5, 0.5 mM DTT, 1 mg/ml lysozyme, 5 U/ml DNaseI and one Complete Protease Inhibitor Cocktail tablet (EDTA free) (Roche Applied Science) per 100 ml lysis buffer) and incubated under stirring at 4°C for 45 minutes. Cells were lysed by sonication (Sonifier, Branson Ultrasonics). Unbroken cells and cell debris were removed by centrifugation at 10,000 g for 10 min at 4°C and the membranes were collected by ultracentrifugation at 30,000 rpm (Beckman Ti45 rotor) at 4°C for 50 min. Membranes were resuspended in 3 ml solubilisation buffer (20 mM sodium phosphate, 300 mM NaCl, pH 7.5, 0.5 mM DTT, and EDTA-free Protease Inhibitor cocktail) per 200 OD_600 nm_ units and stored at −80°C until further use.

### SDS-PAGE and Western Blot analysis

The crude membrane samples were mixed and incubated with reducing NuPAGE® LDS loading buffer (Invitrogen) at room temperature and analyzed on 4–12% NuPAGE® Bis-Tris gels (Invitrogen). SeeBlue® Plus2 Prestained standard (Invitrogen) or BenchMark™ Pre-stained (Invitrogen) were used as protein markers for SDS-PAGE or Western blots, respectively. Proteins were transferred onto nitrocellulose membranes using an iBlot® Gel Transfer system (Invitrogen). Blots were blocked using 1% BSA in TBS-T buffer (20 mM Tris pH 7.5, 100 mM NaCl, 0.05% (v/v) Tween® 20) for 1 hour at room temperature. Membranes were washed 3 times for 10 minutes with TBS-T buffer and then stained with a horseradish peroxidase-labelled His probe (HisProbe™-HRP, Pierce) followed by SuperSignal® West Pico chemiluminescent substrate (Pierce). Signals were detected using a Fluor-S MultiImager (BioRad) CCD camera and associated Quanti One software v.4.2.1.

### Solubilisation and small-scale purification

Based on Western blot results, membranes were chosen for small scale immobilized metal affinity chromatography (IMAC) purification followed by analytical gel filtration runs. After thawing membranes on ice, membranes were solubilised by addition of different detergents (1% final concentration: SDS, FC12, DDM, DM, Triton X-100, LDAO and C_12_E_8_). Samples were kept shaking at 4°C for 60 minutes and were clarified by centrifugation at 100,000 g for 30 minutes at 4°C before loading on IMAC resin. IMAC purifications were performed in disposable Poly-Prep Chromatography Columns (Bio-Rad) (0.8 by 4 cm). 125 µl Ni-NTA agarose beads (Invitrogen) were added to the column and equilibrated with 2 ml wash 1 buffer (20 mM sodium phosphate, 300 mM NaCl, pH 7.5, 30 mM imidazole pH 7.5, 0.5 mM DTT and appropriate detergent. Ni^2+^-beads were incubated with the supernatant of solubilised membranes for 20 minutes at 4°C and subsequently washed two times with 2 ml of wash 1 buffer, followed by 2×2 ml of wash 2 buffer (20 mM sodium phosphate, 300 mM NaCl, pH 7.5, 40 mM imidazole pH 7.5, 0.5 mM DTT and appropriate detergent. Protein was eluted with high concentration of imidazole using 2×0.5 ml elution buffer (20 mM sodium phosphate, 300 mM NaCl, pH 7.5, 400 mM imidazole, pH 7.5, 0.5 mM DTT and appropriate detergent). Elution fractions were analysed on a SDS-PAGE and concentrated to 200 µl for analytical gel filtration.

### Analytical gel filtration

To assess the quality of the purified membrane protein concentrated protein samples were analysed on an analytical gel filtration column (Superdex 200 5/150 GL, GE Healthcare). To guarantee reproducible and reliable gel filtration runs, an ÄKTA Explorer system (GE Healthcare) was coupled to an autosampler, which automatically injected with high-precision 25 µl of protein sample. Analytical gel filtration runs were performed in duplicates in the cold room at a flow rate of 0.2 ml/min in gel filtration buffer (20 mM HEPES pH 7.5, 300 mM NaCl, 0.5 mM DTT with the appropriate detergent). Simultaneous absorption detection at 280 and 400 nm allowed distinction between the target protein and some known IMAC contaminants that also absorb at higher wavelength.

### Large scale over-expression, purification and isotopic labelling

Cultures of 500 ml Terrific Broth (TB) medium in 2.5 L baffled conical flasks were inoculated from a Luria Bertani medium (LB) over night culture and grown identically as the small scale cultures. 16 hours after induction with 0.2 mM IPTG, cells were harvested at 5000 g for 10 minutes and the pellets were stored frozen at −80°C. 1 g of cell pellet (wet weight) was resuspended in 5 ml of lysis buffer and incubated under stirring at 4°C for 45 minutes. Cells were disrupted with an Emulsiflex microfluidizer at 15,000 p.s.i. chamber pressure. Unbroken cells and cell debris were removed by centrifugation at 10,000 g for 10 min at 4°C and the membranes were collected by ultracentrifugation at 30,000 rpm (Beckman Ti45 rotor) at 4°C for 50 min. Membranes obtained from 200 OD_600 nm_ units were resuspended in 3 ml solubilisation buffer (20 mM Na-P, 300 mM NaCl, pH 7.5, 0.5 mM DTT, and EDTA-free Protease Inhibitor cocktail) and solubilised by the addition of 1% dodecyl-ß-D-maltoside (DDM). After 60 min at 4°C, solubilised membranes were clarified by centrifugation at 29,000 rpm for 30 minutes at 4°C before loading on IMAC resin. IMAC beads were incubated with the supernatant for 1 h and loaded in a plastic column (Biorad) and washed with wash buffer containing 20 mM and 40 mM imidazole, respectively. Purified protein was eluted with 250 mM imidazole containing elution buffer, dialysed against 20 mM sodium phosphate, 300 mM NaCl, 5% glycerol, 0.5 mM DTT, 0.03% DDM together with TEV protease over night. Cleaved membrane protein was passed over IMAC beads again and the flow through was collected and concentrated for final gel filtration in 20 mM HEPES, 150 mM NaCl, 5% glycerol, 0.5 mM DTT, 0.03% DDM. Isotopically labelled ^15^N NMR samples were produced using M9 minimal media based on ^15^NH_4_Cl as nitrogen source (Cambridge Isotopes, USA) and supplemented with vitamin mix.

### NMR spectroscopy

All ^1^H and ^1^H-^15^N TROSY-HSQC NMR spectra were acquired with a Bruker Avance III 800-MHz spectrometer using a 5-mm cryoprobe with z-gradients in 20 mM sodium phosphate buffer (pH 7.5), 150 mM NaCl, 0.5 mM DTT, 0.03% DDM, containing 10% ^2^H_2_O at different temperatures. Protein concentration was 0.4 mM for all measurements. A standard pulse excitation sequence using the WATERGATE element [50] and pre-saturation was used for one-dimensional proton spectra. Each proton spectrum was recorded with 1024 transients. All two-dimensional spectra were recorded using a sensitivity enhanced version of the TROSY-HSQC approach [51]. Each TROSY-HSQC spectrum was recorded within 2 h 40 min. All ^1^H spectra were processed and analysed by Topspin 2.1. ^1^H-^15^N spectra were processed by NMRPipe [52] and analysed by NMRView [53].

### Equilibrium CD spectroscopy

All experiments were performed at 20°C in 20 mM sodium phosphate buffer (pH 7.5), 150 mM NaCl, 0.5 mM DTT, 0.03% DDM. CD spectra were recorded with a JASCO J600A spectropolarimeter (0.1 cm cell length, 10 µM protein concentration, 1 nm bandwidth) and corrected for the buffer contributions. Due to the high lipid content in the protein preparation Far UV-CD spectra could only be recorded till 196 nm. Corrected CD spectrum was analyzed with the online software package CDPro.

### Thermal stability studies by DSLS

Thermal stability of synaptogyrin 1 was studied by differential static light scattering (DSLS) using Stargazer (Harbinger Biotechnology and Engineering Corporation, Markham, Canada). Protein stability is measured by monitoring protein aggregation upon protein denaturation with increasing temperature at 600 nm. Synaptogyrin 1 (construct 5) at 0.3 mg/ml in a 50 µl-volume in a clear-bottom 384-well plate (Nunc) was heated from 25 to 80°C at 1°C/min. Wells were covered with 50 µl mineral oil to minimize evaporation. Protein aggregation was monitored and analysed as described [44]. To study the influence of different buffers (pH 4–9), salts (0–500 mM NaCl, 0–300 mM KCl, 0–20 mM CaCl_2_), detergents and additives (as cholesteryl hemisuccinate and lipids) on the aggregation behavior of synaptogyrin 1, purified protein was diluted from a stock solution (10 mg/ml) into the new buffer condition.

### Electron microscopy

The synaptogyrin preparations were imaged by negative staining as follows. Aliquots of 5 µl protein diluted to 20 µg/ml were adsorbed on the carbon-film coated copper grids, washed with 10 droplets of pure water and subsequently stained with 2% uranyl-acetate. Images were recorded using a Philips CM100 TEM operated at 100 kV.

### Crosslinking and limited proteolysis

Crosslinking experiments were performed at room temperature on crude membranes and purified protein in the detergents DDM and Triton X-100 according to the manufacturer instructions (Invitrogen) using the crosslinkers DMS (Dimethyl suberimidate-2HCl), DSS (Disuccinimidyl suberate) and glutaraldehyde. The crosslinking reaction was quenched after different time points upon addition of 200 mM Tris buffer. Samples were subsequently analyzed on SDS-PAGE and Western blotting using a horseradish peroxidase-labelled His probe. Stability of synaptogyrin 1 against proteolytic degradation was performed on DDM purified protein in the presence of different concentrations of chymotrypsin (chymotrypsin: protein ratio: 1∶100–1∶10 000). Reaction was stopped after different time points by heating the sample and subsequent analysis on SDS-PAGE.
